# A Case of Tetanus Cured by Hypodermic Injections of Cocaine

**Published:** 1887-11

**Authors:** 


### A Case of Tetanus Cured by Hypodermic Injections of Cocaine.

Dr. M. Lopez reports the following case in *El Genio Medico-Quirurgico* for February 7, 1887: M. G., 50, laborer, after working in the wet and cold, complained of rheumatic pains of back and limbs. Three days later he had marked opisthotonus and painful cramps, and all the symptoms of idiopathic tetanus. Chloral hydrate and morphine were prescribed. For three days the patient was kept under the influence of these drugs, with the result that the pain was lessened, but the muscular rigidity and cramps increased. He now became unable to swallow, and death seemed imminent. Morphine was injected hypodermically, but was followed by no amelioration of the symptoms. Three syringefuls of the mixed solutions of morphine and cocaine (each five per cent.) were then injected. The effect was immediate. After two hours he could move the limbs, turn in bed, and open his mouth. On the next day he was going on well; slight trismus and stiffness of neck remained. On both sides of the neck, and at the angle of the jaw, a fourth part of a syringeful of the same solution was injected. On the next day all the symptoms had disappeared. The patient rapidly regained strength, and in a week's time resumed work.—*London Medical Record.*

